# *Listeria monocytogenes*: A Continuous Global Threat in Ready-to-Eat (RTE) Foods

**DOI:** 10.3390/foods14213664

**Published:** 2025-10-27

**Authors:** Jamyang Yangchen, Dipon Sarkar, Laura Rood, Rozita Vaskoska, Chawalit Kocharunchitt

**Affiliations:** 1 Centre of Food Safety & Innovation, University of Tasmania, Private Bag 54, Hobart, TAS 7005, Australia; dipon.sarkar@utas.edu.au (D.S.); laura.rood@utas.edu.au (L.R.); 2 Victual Pty Ltd., Level 10, 100 Arthur Street, Sydney, NSW 2060, Australia; 3 Agriculture and Food Research Unit, Commonwealth Scientific and Industrial Research Organisation, 671 Sneydes Road, Melbourne, VIC 3030, Australia; rozita.spirovskavaskoska@csiro.au

**Keywords:** food safety, food recall, regulatory requirements, microbiological criteria, antimicrobial intervention strategies

## Abstract

*Listeria monocytogenes* is a significant foodborne pathogen associated with high rates of hospitalization and death, especially among vulnerable populations. Despite established regulatory standards and available antimicrobial intervention strategies, *L. monocytogenes* remains as a pathogen of concern in ready-to-eat (RTE) foods. This ultimately can lead to food recalls or listeriosis outbreak, highlighting its ongoing risks to food safety and public health. This review consolidates publicly accessible surveillance case counts and recall data of *L. monocytogenes* contamination from Australia, Europe, Canada, and the United States to assess the contamination trends in the RTE food supply chain. It also evaluates the effectiveness of antimicrobial intervention strategies, including both those currently implemented in industry and those that have been studied as potential interventions but are not yet widely adopted. Key factors affecting the efficiency of those strategies are identified, including food matrix composition, water activity (a_w_), fat content, and strain variability. Emerging multi-hurdle technology that integrates physical, chemical, and biological antimicrobial interventions are highlighted as promising approaches for maintaining both food safety and product quality. It also outlines the role of quantitative microbial risk assessment (QMRA) as a decision-support tool to select appropriate control strategies, predict recall risk and guide evidence-based risk management. Future research directions are proposed to expand the application of QMRA in managing recall risks throughout the RTE food supply chain due to *L. monocytogenes*.

## 1. Introduction

*Listeria monocytogenes* is a serious foodborne pathogen of public health concern, associated with listeriosis. Though listeriosis is rare, it is a serious illness with high hospitalization and mortality rates, particularly in vulnerable populations, such as the elderly, pregnant women, neonates, and immune-compromised individuals [1,2,3]. Globally, the World Health Organization (WHO) estimated an incidence of 0.2 cases per 100,000 population in 2010 [4,5]. In the United States (US), the rate of listeriosis is 0.24 cases per 100,000 population [6,7]. In Australia, the most recent data on the number of listeriosis cases per annum is from 2023, which was around 0.33 cases per 100,000 population per year [8,9]. In 30 countries of the European Union and European Economic Area (EU/EEA), the rate was 0.67 cases per 100,000 population in 2023 [10].

*L. monocytogenes* is ubiquitous and present in the environment, water, and raw foods of both plant and animal origin. It can persist in harsh conditions and thrive at refrigeration temperatures [11,12,13]. The bacterium’s ability to develop biofilms on food contact surfaces exacerbates sanitation challenges and heightens the danger of cross-contamination in food manufacturing and processing [13,14]. Ready-to-eat (RTE) foods such as pre-prepared salads, cold RTE chicken, deli meats, pasteurized milk, soft cheese, and smoked salmon are consumed without further cooking or pathogen inactivation steps [15,16]. This makes RTE foods a significant vehicle for listeriosis transmission, and they are considered high risk in terms of food safety [15,17]. The prevalence of *L. monocytogenes* in the food supply chain has led to numerous food recalls and listeriosis cases globally. Indeed, *L. monocytogenes* contamination was a leading cause of microbiological food recalls in Australia, Europe, the US, and Canada between 2012 and 2022 [18,19,20,21]. The recall data from the US and compliance data in Australia for both locally produced foods and imported foods also indicate that RTE foods are mostly recalled because of *L. monocytogenes* contamination [18,22,23,24,25,26]. These trends emphasize the need for robust, farm-to-fork control strategies to proactively manage *L. monocytogenes* contamination risks across the RTE food supply chain.

In food processing environments, the control of *L. monocytogenes* relies on stringent sanitation practices, environmental monitoring programs, and the implementation of robust preventive controls. Facilities adopt Good Manufacturing Practices (GMPs) and Hazard Analysis and Critical Control Point (HACCP) systems tailored to minimize contamination risks, particularly in high-risk areas, such as slicing, packaging, and post-lethality zones [27]. Preventive strategies include the design of hygienic equipment, strict zoning between raw and processing areas, air handling systems to prevent cross-contamination, and regular cleaning using validated sanitizers [28,29]. Environmental sampling plans focus on food contact and non-contact surfaces, allowing for early detection and response to *Listeria* species [29]. Additionally, biofilm control strategies, staff hygiene training, and raw material control are key components of ongoing risk mitigation [27]. Despite these practices, persistent contamination and the survival of *L. monocytogenes* in harborage sites continue to challenge food processors, reinforcing the importance of continuous verification and improvement of control measures.

Currently, the risk of *L. monocytogenes* in RTE foods is primarily managed through strict regulatory standards. These include microbiological limits and monitoring requirements of products and processing environments, along with disease surveillance measures, such as mandatory notification of listeriosis cases by general practitioners and laboratories to public health authorities [30,31]. Alongside regulatory compliance, food producers implement a range of antimicrobial intervention strategies such as thermal or non-thermal treatments and the use of biological agents, such as nisin or chemical preservatives, to control *L. monocytogenes*. However, each intervention strategy comes with specific advantages and limitations. Their effectiveness can vary depending on the food matrix, application method, and processing conditions. In some cases, interventions may negatively impact product quality or consumer acceptability. Strategies that maintain sensory quality and consumer appeal often show limited effectiveness when used individually and are generally more effective when applied in combination. As a result, there is a need to explore novel or integrated intervention strategies. Rather than relying solely on time-consuming and resource intensive experimental trials, quantitative microbial risk assessment (QMRA) frameworks offer a promising solution, enabling science-based evaluation of different interventions in reducing contamination risk. For example, the FAO/WHO QMRA for *L. monocytogenes* in RTE foods modeled how varying levels of contamination and intervention scenarios affected the probability of illness, providing a science based foundation for establishing microbiological criteria [32].

While numerous reviews have addressed *L. monocytogenes* prevalence, virulence, or individual control measures, there is limited synthesis that explicitly links recall, outbreak, and surveillance data with the effectiveness and limitations of antimicrobial interventions used in RTE foods. This review bridges that gap by integrating publicly available food safety data, including compliance testing, prevalence surveys, and recall notifications with a critical evaluation of intervention strategies applied across various RTE food categories. In addition, while QMRA has traditionally been used for predicting illness outcomes, this review highlights emerging applications of QMRA in estimating shelf-life and recall risk, which are particularly underexplored. This integrative approach not only strengthens the understanding of *L. monocytogenes* risk in RTE foods but also emphasizes the need for data-driven, industry-relevant decision support tools that align with evolving regulatory frameworks and consumer safety demands. By addressing these gaps, the review offers a novel perspective on improving food safety management from both a regulatory and operational standpoint.

This review aims to synthesize publicly available outbreak, surveillance, and recall data alongside widely used and emerging intervention strategies to provide a more comprehensive understanding of *L. monocytogenes* risk in RTE food. Hence, the objectives of this review are to (i) analyze the prevalence and risk of *L. monocytogenes* in RTE foods in Australia, with comparisons to trends in Europe, Canada, and the US; (ii) examine global antimicrobial intervention strategies for controlling *L. monocytogenes* in RTE foods and identify its limitations; and (iii) highlight the emerging opportunities for future research in the field of QMRA to address the risk of *L. monocytogenes* in RTE foods.

## 2. *Listeria monocytogenes*

*L. monocytogenes* is a Gram-positive, non-spore forming, facultatively anaerobic rod [33], and it is ubiquitous in nature [34]. It is present in soil, water, decomposing plants, animal excrement, and food processing environments [34]. The organism exhibits notable environmental adaptability. It can grow at temperatures from 4 to 45 °C, survives a wide pH range (4.7–9.2), and requires moderate water activity (a_w_ > 0.92) [34]. This allows it to persist in food processing environments, survive various control strategies, and proliferate in food products [35,36]. Another significant factor in the persistence of *L. monocytogenes* in food environments is its ability to form biofilms on equipment and food contact surfaces [13,14,37]. These biofilms provide protection to the bacterial cells against environmental stressors, such as desiccation, nutrient limitation, and exposure to antimicrobial agents [38,39,40]. Moreover, they present a major challenge for sanitation efforts, as cells embedded within biofilms can survive standard cleaning and disinfection procedures [41,42]. This resilience makes *L. monocytogenes* particularly difficult to eliminate once established in processing environments. In fact, research has shown that certain *L. monocytogenes* strains are capable of long-term persistence in food facilities, surviving for several months or even years [13,43]. A study by Leong et al. [44] also reported that four out of seven Irish food processing facilities (isolated ≥ 6 months apart) had persistent *L. monocytogenes* strains (belonging to serotype 1/2a, 1/2b, 1/2c, 4b) in both environmental and food samples.

Potential sources of *L. monocytogenes* to food processing plants can be introduced from raw materials, food handlers, water, or equipment. However, the primary sources of contamination are often food handlers or equipment [13,40,43,44,45,46]. Additionally, Samelis and Metaxopoulos [47] concluded that post-processing contamination, particularly in areas such as cooked meat slicing, was a major issue. Their research indicated that sliced vacuum-packed cooked meats and country style sausages were more contaminated (6.7–10%) in comparison to non-sliced cooked meats in which *L. monocytogenes* was not detected [47]. Similarly, Zhang et al. [48] investigated *L. monocytogenes* contamination in two RTE food plants in Shanghai, China, and reported that 12.1% of 239 samples were positive, including 21 samples from plant A. In plant A, *L. monocytogenes* was primarily isolated from pickling, cooling, and packaging areas [48]. In another study, Muhterem-Uyar et al. [49] analyzed 2242 food processing environment samples collected from twelve facilities across six European countries (Austria, Ireland, Spain, Slovakia, Greece, and Romania) over one year. They outlined three contamination scenarios: (i) occasional contamination at the junction between raw material reception and hygienic areas; (ii) localized contamination hotspots in hygienic processing zones; and (iii) extensive contamination across the entire processing environment. Similarly, Ho et al. [50] conducted a three-year longitudinal study in a farmstead dairy processing facility and found that *L. monocytogenes* contamination was largely confined to drains and floors, with persistent PFGE subtypes (DUP-1052A in the processing plant; DUP-1039A in the milking parlor), and a subtype detected only once in each area (DUP-1030A), consistent with sporadic contamination. These findings emphasize site specific persistence and cross-contamination pathway, indicating the need to control post-lethality exposure (especially slicing lines), prioritize hygienic design and sanitation of wet niches (such as drains/floors), and maintain targeted environmental monitoring capable of detecting both persistent and sporadic events.

### 2.1. Importance of L. monocytogenes Biofilms

The ability of *L. monocytogenes* to form biofilms is a key factor in its long-term survival and persistence in food processing environments. Microbial biofilms are composed of microbial cells attached to a surface and embedded in an extracellular matrix. Development of biofilms typically proceeds through (1) attachment, (2) cell proliferation and production of extracellular polymeric substances (EPS), (3) early development of biofilm architecture, (4) maturation, and (5) dispersion [51,52,53]. The early stages (step 1–3) are facilitated by motility and adhesion factors; however, during maturation, cells lose motility. Matrix build-up and high cell density provide protection from antimicrobials and disinfectants, increasing tolerance and enabling persistence [39,40].

*L. monocytogenes* forms biofilms on a variety of food and non-contact food surfaces commonly found in RTE facilities (e.g., stainless steel, rubber, plastic, and even conveyor belts and drain covers) [37,43]. Persistent contamination has been reported on stainless steel, with adhesion enhanced at low temperatures [40,46]. When *L. monocytogenes* cells are embedded in biofilms, a subpopulation can enter a reversible dormant state (viable but non-culturable, VBNC), complicating detection because culture methods fail to recover these cells [53,54,55,56].

*L. monocytogenes* detection in processing environments should draw on learnings from in-product testing that has demonstrated that molecular-based methods are more efficient in detection contamination than culture-based methods. For instance, Panera-Martínez et al. [57] detected *L. monocytogenes* in 50.0% by culture (isolation on OCLA and confirmation by conventional PCR) and 66.7% by q-PCR (concentrations ranged 2.40–5.22 log_10_ CFU/g (total) and <2.15–3.93 log_10_ CFU/g viable *L. monocytogenes*) on raw poultry samples. v-PCR showed 100% sensitivity, 66.7% specificity, and 83.3% efficiency vs. the culture gold standard (kappa coefficient = 0.67), supporting v-PCR for detecting viable cells that culture may miss [57]. Extending this approach to chicken meat, Panera-Martínez et al. [58] reported *L. monocytogenes* in 75.0% of samples; v-PCR detected viable cells in 30.8% of samples versus 19.2% by plating. Mean counts (log_10_ CFU/g) were 4.01 (total), 3.21 (viable), 1.00 (viable-culturable), and 3.20 (VBNC), with VBNC cells comprising 15.66% of total and 99.38% of viable cells [58]. This evidence suggests that culture-dependent testing fails to capture the full extent of *L. monocytogenes* contamination and risk.

### 2.2. Food Safety Risk of L. monocytogenes

*L. monocytogenes* presents significant challenges to food safety and public health authorities worldwide due to its ability to cause listeriosis [1]. Human infection typically occurs through the consumption of contaminated food [59], and the incubation period can range from a few weeks to three months [32]. Additionally, *L. monocytogenes* can transiently reside in the human intestinal tract, with 2–10% of the population being asymptomatic carriers [60].

Listeriosis presents in two clinical forms: non-invasive and invasive form [2]. Non-invasive listeriosis only affects healthy individual and causes mild gastrointestinal disorder, which resolves without any treatment [2]. On the other hand, invasive listeriosis is severe and affects high-risk population groups, notably the elderly, neonates, pregnant women, and immune-compromised individuals [33,61,62]. The symptoms include septicemia, meningitis, and even miscarriage and death [1]. These vulnerable groups have reduced immune defenses, making them more susceptible to invasive infections [61]. In healthcare settings, the risk is amplified because of the presence of a large number of highly vulnerable individuals. For instance, during the 1999 outbreak in Finland linked to butter, 15 of the 25 affected patients were severely immunocompromised and had prolonged hospital stays, with 60% of cases occurring in a single tertiary hospital [63].

The severity of listeriosis is further emphasized by the low infectious dose (ID_50_) required to cause illness in susceptible individuals. The ID_50_, defined as the dose needed to infect 50% of a population, varies depending on host susceptibility and the characteristics of the food matrix [62,64]. In animal models such as mice, the oral ID_50_ for *L. monocytogenes* has been reported to be around 6.4 log_10_ CFU [65]. However, for high-risk human populations, a considerably lower dose may be sufficient to cause illness [32,63]. This was evident during an outbreak in Finland, where butter samples contained 0.7–1.8 log_10_ CFU/g of *L. monocytogenes* (one sample reaching 4.0 log_10_ CFU/g), yet still caused illness in highly vulnerable patients [32,63]. Certain food matrices, particularly high-fat products like butter or cheese, can enhance bacterial survival in the gastrointestinal tract, thus reducing the effective infectious dose [66]. Additionally, host-related factors, such as gastric acid suppression caused by medications like antacids or proton pump inhibitors, can impair natural barriers to infection and further increase susceptibility [67].

Beyond differences in host susceptibility and infectious dose, *L. monocytogenes* exhibits genetic diversity that influences its virulence, ecological niches, and persistence in food processing environments. *L. monocytogenes* is classified into 4 genetic lineages and 13 serotypes, out of which 1/2a, 1/2b, 1/2c, and 4b are mostly associated with listeriosis in humans [33,68,69,70,71]. Lineage I strains (serotype 1/2b, 3b, 3c, and 4b), particularly serotype 4b, are associated with major outbreaks and severe invasive disease, while lineage II strains (serotype 1/2a, 1/2c, and 3c), especially serotype 1/2a, are more prevalent in environmental and food isolates and are often associated with sporadic cases [33,69,71,72]. Lineage III (serotype 4a, 4b, and 4c) and IV strains (serotype 4a, 4b, and 4c) are rarely isolated from clinical cases but are predominately isolated from animal sources [72,73]. This distribution highlights that certain lineages and serotypes may have an enhanced capacity to persist in processing environments and cause listeriosis, reinforcing the importance of molecular surveillance and targeted intervention strategies in the food industry.

### 2.3. Global Trends and Impact of Listeriosis

In 2020, the WHO Foodborne Disease Burden Epidemiology Reference Group (WHO/FERG) estimated that foodborne listeriosis caused around 14,169 illnesses, 3175 deaths, and 118,340 DALYs worldwide based on the data compiled from 45 member states to generate global estimates [5,74]. Among these cases, 79.3% were non-invasive, while 20.7% were invasive, with fatality rates estimated at 14.9% and 25.9%, respectively [5,74]. Given the potential severity of illness and the high-risk nature of certain population groups, ongoing surveillance of listeriosis incidence and *L. monocytogenes* prevalence in the food supply chain is critical for effective food safety management. However, comprehensive surveillance data is only available for Australia and Europe, making these regions the primary focus for current trend analysis.

**Figure 1:**
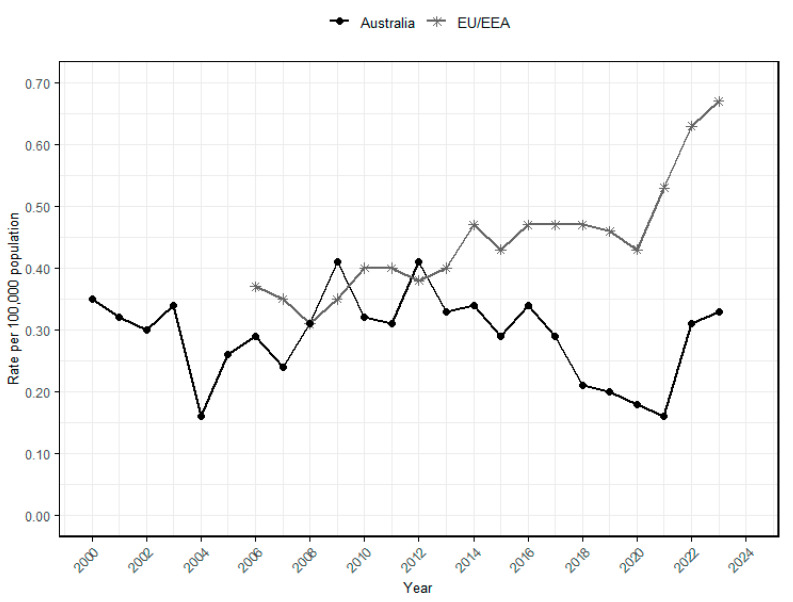
Annual listeriosis incidence rates per 100,000 population in the European Union and European Economic Area (EU/EEA) and Australia. The surveillance data of Australia (2000–2023) was obtained from the National Notifiable Diseases Surveillance System (NNDSS) database [8,77,78], and annual population estimates were extracted from Australian Bureau of Statistic [79]. The listeriosis rates for the EU/EEA were obtained from the European Centre for Disease Prevention and Control’s (ECDC) Annual Epidemiological Reports. Starting in 2020, the rate from the United Kingdom was excluded from this report [10,80,81,82,83,84,85,86,87,88,89,90,91,92,93].

In Australia, the National Notifiable Diseases Surveillance System (NNDSS) records the incidence of communicable diseases, including foodborne infections like listeriosis. Surveillance data spanning 23 years (2000–2023) indicate a fluctuating trend in the incidence rate of listeriosis (Figure 1). Between 2000 and 2003, the rate remained relatively high (0.30–0.35 cases per 100,000 population) before declining in 2004 (0.16 cases per 100,000 population). Subsequently, the incidence rate generally increased and remained relatively stable through the 2010s (around 0.25–0.30 cases per 100,000 population). The rate then decreased during 2020–2021 (0.16–0.18 cases per 100,000 population), likely due to reduced surveillance and reporting during the COVID-19 pandemic, in addition to possible changes in exposure patterns. Factors such as reduced consumption of RTE foods because of limited dining out during lockdowns and improved hygiene practices (e.g., mandatory handwashing) may have contributed to the decline [75,76]. The rate rose again in 2022 and 2023 (0.31 and 0.33 cases per 100,000 population, respectively), indicating the continuing public health importance of *L. monocytogenes* in Australia.

In contrast, the European Union and European Economic Area (EU/EEA) exhibited a generally increasing trend in incidence rates over 18 years (2006–2023), rising from around 0.35 cases per 100,000 population in 2006 to 0.46 cases per 100,000 population until 2019. A minor dip occurred in 2020 (0.43 cases per 100,000), coinciding with the COVID-19 pandemic and the withdrawal of the United Kingdom from EU surveillance. However, it gradually increased to about 0.67 cases per 100,000 population in 2023. This long-term upward trajectory highlights that *L. monocytogenes* remains a persistent food safety and public health concern in Europe.

Listeriosis has emerged as a major global public health issue, imposing substantial economic costs and social impacts. In Australia, the total annual cost of listeriosis was estimated to be around AUD 78.4 million annually in terms of medical expenses, lost productivity, premature mortality, and hospitalization [94]. This is similar to the US, where the cost is estimated to be around USD 2.8 billion in 2013, equivalent to AUD 4.3 billion [95]. Surveillance data from Australia and Europe, along with the economic burden in both Australia and the US, demonstrate that *L. monocytogenes* represents a significant public health concern not only in Australia but also in major countries like the US and Europe.

## 3. Ready-to-Eat (RTE) Foods

RTE foods are products that do not undergo any listericidal treatment and are consumed in the same state as they are sold [15]. Nuts in the shell, raw fruits, and vegetables intended for washing or peeling by consumers are not considered RTE foods [15]. Similarly in the EU, RTEs are foods that can be described as those consumed without the need for additional processing to meet safety and microbiological criteria [96]. They belong to a large heterogenous category of foods and are convenient for today’s lifestyle because they do not require cooking or further preparation [27].

RTE foods are broadly classified as high-risk or low-risk based on their ability to support the growth of *L. monocytogenes*. This classification is adopted by major food safety authorities, including Food Standards Australia New Zealand (FSANZ), the Codex Alimentarius Commission, the European Commission, and Health Canada. For example, FSANZ classifies RTE foods into two categories depending on whether they support *L. monocytogenes* growth, applying stricter limits (absence in 25 g) for higher-risk foods. Likewise, Canada uses a similar approach, classifying RTE foods into Category 1 (able to support the growth) and Category 2 (not supporting the growth) of *L. monocytogenes*, with stricter compliance limits for Category 1 products [97]. This classification is based on the intrinsic properties of RTE foods, mainly pH and water activity (a_w_). Table 1 provides a comparison of international criteria and examples of high-risk and low-risk RTE food categories. High-risk RTE foods are particularly susceptible to contamination during post-processing handling [98].

Among RTE food categories, deli meats, smoked seafood (e.g., cold-smoked salmon), and soft cheeses (e.g., Brie, Camembert, Queso Fresco) are consistently considered high-risk for *L. monocytogenes* contamination. These products share several risk-enhancing characteristics that relate to their food matrix properties, post-processing contamination, and intervention strategies. In relation to food matrix properties, they typically have intrinsic properties, such as high water activity (a_w_ > 0.92) and neutral pH (≥5.0), supporting the survival and growth of *L. monocytogenes* [99,100]. Intervention-wise, many of these products do not receive a listericidal treatment after packaging, making them particularly susceptible to post-processing contamination during slicing, handling, or repackaging. Physical interventions are prohibitive because they change the nature and properties of the product, while chemical intervention are restricted to very few approved additives or processing aids such as sodium lactate and sodium diacetate that prevents microbial growth [101,102,103]. Biological interventions (e.g., phages, bacteriocins) are likewise constrained to a limited set of approved uses [100,104,105,106,107,108]. Finally, these product groups are prone to post-processing contamination due to being handled and packaged into formats convenient to the consumer, such as slices or pieces, thus exposing their surface to contamination through knives and slicers. For example, cold-smoked salmon is not heat-treated after smoking, and RTE meats like ham or turkey slices are frequently re-contaminated at various stages of production and distribution [13,108,109,110,111]. *L. monocytogenes* can grow by 1.3 log_10_ CFU/g at 4 °C over 28 days in smoked salmon [112]. In a related real-world example, Stessl et al. [113] investigated a sporadic listeriosis cluster in Austria during 2015–2017 across three Austrian provinces and, in a subsequent five-year environmental monitoring program, identified a schnitzel-sorting machine as the likely contamination source.

## 4. Significance of *L. monocytogenes* in RTE Supply Chain

The susceptibility of RTE foods to *L. monocytogenes* contamination is well established due to their minimal processing prior to consumption and extended refrigerated shelf life [114]. Analysis of food recall and compliance data, with particular attention to RTE foods, reinforces this vulnerability by providing concrete evidence of contamination trends across product types and regions. When food products exceed regulatory thresholds for *L. monocytogenes* (further outlined in Section 5.1), regulatory actions, such as mandatory reporting and product recalls, are initiated to protect public health. In Australia, one such measure is the mandatory reporting of *L. monocytogenes* detection during routine or quality testing. Commercial laboratories and food businesses are required to notify the food safety authority within 24 h of detection, and reporting requirements differ from state to state [115]. Other key measures are the recall and withdrawal of non-compliant food products. A food recall is the process of removing food products from sale, distribution, and consumption to address safety risks to consumers [116]. Product withdrawal is an action taken to remove food products from sale as a result of quality defect or as a precautionary measure when further investigation is needed to analyze the potential health risks to consumers [116]. To identify the types of RTE foods most commonly associated with *L. monocytogenes* contamination, this section looks into the publicly available food recall data, primarily from the US and Australia due to data unavailability in other countries. Detection during production and resulting withdrawals typically trigger corrective actions, such as sanitation interventions, process modifications, or reformulation [16,97,117], while recalls from the market are often followed by root cause investigations, increased monitoring, and regulatory scrutiny to prevent recurrence [118,119].

### 4.1. National and International Food Recall Data

National and international food recall data provide valuable insights into the continued risk posed by *L. monocytogenes* in the food supply, particularly in RTE foods. Table 2 summarizes publicly available food recall data from Australia, Canada, and the US; for the EU, the iRASFF notification count is included. The recall data from the EU could not be obtained, as the EU does not publish consolidated EU wide recalls figures. The EU’s Alert and Cooperation Notification (ACN), shared through the iRASFF platform, records events by notification type rather than recall counts. The ACN covers the following aspects: (1) non-compliances with possible health risk; (2) non-compliances without health risk; (3) suspicions of fraud; (4) non-compliant consignments of plants and plant products, as well as issues related to contingency plans for plant pests and other plant health concerns; and (5) non-compliances and fraud suspicions related to companion animals and animal welfare. Notifications are classified as alert, information for attention, information for follow-up, border rejection, or news. Measures for outbreaks/food poisoning are recorded in in the follow-up section of the ACN annual reports [120].

Across countries, recalls due to microbiological hazards account for 19.4–38.5% of total recalls (Australia, Canada, and the US), and recalls due to *L. monocytogenes* represent 31.1–43.2% of microbiological recalls (Australia, the US). For the EU, notifications due to microbiological hazards constitute 55.7% of all notifications, and *L. monocytogenes* account for 10.0% of microbiological notifications. While some data reflect all food types, RTE foods, particularly deli meats, pre-packaged salads, dairy products, and seafood, consistently emerge as high-risk categories within these recalls [18].

**Figure 2:**
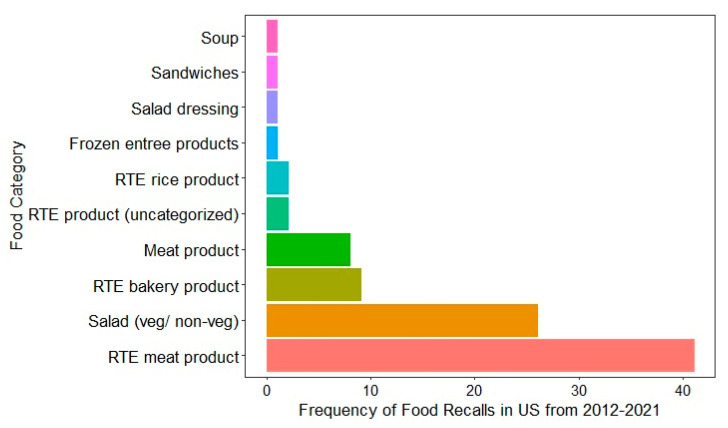
Food categories associated with food recall due to *L. monocytogenes* contamination in the US from 2012 to 2021 [123].

Recent food recall data from the US (2012–2021) highlights that the types of food products most commonly recalled due to *L. monocytogenes* contamination are RTE meat products, as shown in Figure 2 [18]. These products, such as deli meats, hot dogs, and sausages, are susceptible to contamination during processing, packaging, and handling, especially in facilities with inconsistent hygiene protocols. For instance, a notice of suspension was issued by the USDA FSIS to Boar’s Head Provisions, Co., Inc., to revoke the federal marks of inspection and suspend the operations of RTE products at Boar’s Head Provisions, Co., Inc., Virginia, due to unsanitary conditions of the processing unit [122]. The second most recalled food was pre-packaged salads, both vegetarian and non-vegetarian [18]. Comparable product-specific recall data were not publicly available for Australia, Canada, or the EU, where only aggregated recall figures or broad food categories are reported without specifying the pathogen responsible. This data gap limits cross-country comparisons and emphasizes the importance of improved transparency and harmonization in food recall reporting. The trends observed in the US emphasize the vulnerability of RTE products to *L. monocytogenes* and highlight the need for stringent monitoring and control measures in the production and handling of high-risk foods, such as RTE meats, dairy, fish, and fresh produce. This persistent association with contamination emphasizes the ongoing challenges in managing *L. monocytogenes* in RTE foods.

### 4.2. National Compliance and Prevalence of L. monocytogenes in RTE Foods

**Figure 3:**
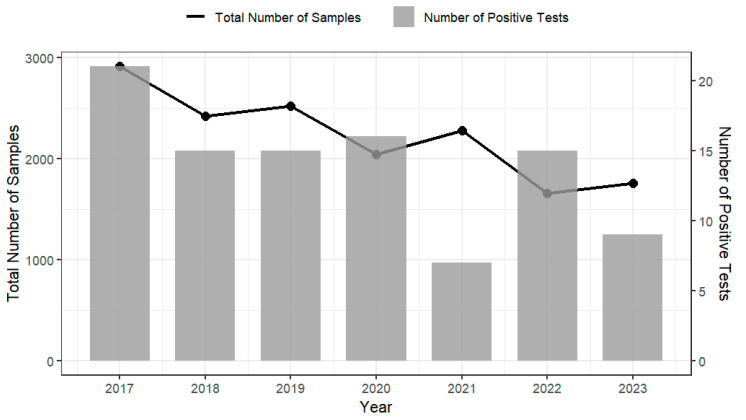
Annual testing results of imported RTE foods for *L. monocytogenes* by the Australian Department of Agriculture, Fisheries and Forestry between 2017 and 2023 [22]. The left *y*-axis and black line represent the total number of samples tested each year; the right y-axis and grey bars represent the number of samples positive for *L. monocytogenes*.

Analysis of testing data on imported products from the Australian Department of Agriculture, Fisheries and Forestry (2017–2023) indicates that products such as cheese, RTE seafood, and processed meats demonstrated consistently high compliance with regulatory requirements for *L. monocytogenes*, with annual compliance rates generally exceeding 99% [22]. However, despite these high compliance levels, non-compliant foods were recorded each year (see Figure 3), indicating that *L. monocytogenes* remains a manageable yet persistent risk. This trend is mirrored in the prevalence surveys of domestically produced RTE foods conducted by FSANZ and relevant health departments of ACT and NSW on various domestically produced RTE foods [23,24,25,26]. These surveys report *L. monocytogenes* prevalence rates ranging from 0.4% to 3.9% across different food types and regions (Table 3). While the data suggest a possible decline in contamination levels over time, the continued detection of *L. monocytogenes* demonstrate the importance of maintaining robust food safety controls and active surveillance throughout the RTE food supply chain. The compliance testing graph (Figure 3) reports regulatory non-compliance at the border (i.e., results assessed against microbiological criteria), while the prevalence survey (Table 3) reports positivity from targeted surveys of specific products and settings. These sources differ in sampling frame (imports/consignments vs. retail/food-service), design (rate-based regulatory screening vs. study-specific surveys), product scope (mixed imported RTE foods vs. single categories, like cooked prawns, sliced RTE meats, sandwiches), period (2017–2023 vs. 2003–2016), definitions (non-compliant vs. positive), methods and detection limits, and sample size (with some survey denominators marked NA). Due to these differences, the lower non-compliance rates (0.3–0.9%) from border compliance testing and the higher survey positivity (0.4–3.9%) are not directly comparable; cross-stream inferences are indicative only and should be interpreted with caution.

## 5. Current Intervention Strategies

### 5.1. Risk-Based Regulatory Framework

In recent years, food safety management has increasingly adopted a risk-based approach, grounded in the principles of risk analysis [124]. The risk analysis framework comprises three key components: risk assessment, risk management, and risk communication [125]. This framework enables evidence-based decision making by evaluating hazards and identifying appropriate control measures. Within this framework, risk assessment plays a central role in evaluating potential hazards in the food supply [124,125]. For microbiological hazards such as *L. monocytogenes*, quantitative microbial risk assessment (QMRA) is widely used to integrate data on contamination levels, consumption patterns, and dose–response relationships to estimate the probability of illness [126,127]. This scientific approach enables regulators to establish food safety limits based on both the likelihood of contamination and the severity of health outcomes.

A QMRA conducted by the Food and Agriculture Organization (FAO) and the World Health Organization (WHO) evaluated the risk of *L. monocytogenes* in various RTE food categories [128]. This international risk assessment has been widely referenced and used as a foundational document for guiding food safety policies and microbiological criteria for the food product. The outcomes of these assessments support the development of regulatory requirements tailored to specific regions, as shown in Table 4. To comply with such standards, food businesses may be required to demonstrate whether their products support the growth of *L. monocytogenes* [16,27,96,97,99]. This is often carried out through challenge testing, which assesses the pathogen’s behavior under specific storage and handling conditions. The results of such studies help determine whether additional control measures are necessary to ensure product safety throughout shelf life and to meet regulatory compliance for *L. monocytogenes*.

### 5.2. Antimicrobial Intervention Strategies to Meet Requirements

To mitigate *L. monocytogenes* in RTE foods, food businesses utilize various intervention strategies that can be broadly classified into physical, chemical, and biological methods. These strategies aim to either eliminate the pathogen or suppress its proliferation to guarantee food safety for the entire shelf life [130]. Table 5 summarizes the different antimicrobial intervention strategies, including their type, brief descriptions, product types applied, efficacy and limitations. The studies included in the table were selected based on their relevance in demonstrating the efficacy of intervention strategies against *L. monocytogenes* in RTE foods. It is important to note that the efficacy of interventions varies with both product and process conditions (see Table 5). For RTE deli meats, a 3 log_10_ reduction may require 266 min at 55 °C, whereas high-pressure processing of cooked ham at 504 MPa for 3 min can achieve a 4 log_10_ reduction. The influence of product formulation is also seen; even at the same temperature, the time to a 7 log_10_ reduction differs (at 68.9 °C, it is 8.1 min for lean sausage and 8.4 min for fat sausage).

Beyond product and process effects, efficacy also varies with environmental and food-related stresses (acid, salt concentration, temperature) and strain/serotype variability to such stresses [131,132,133,134,135,136]. For instance, Zhou et al. [133] observed shorter lag times for clinical strains under 0.50% NaCl/0.04% bile salt, and Myintzaw et al. [135] reported better low-temperature growth among clinical isolates; serotype 4b (lineage I) has been reported as more osmotolerant than other serotypes. Thus, one-size-fits-all controls are inadequate, and this strain/serotype variability is not reflected in Table 5 (full discussion in Section 5.2.1 Challenges and Considerations in Implementing Intervention Strategies for details).

Physical interventions include both thermal (e.g., conventional pasteurization, infrared heating) and non-thermal treatments (e.g., high-pressure processing, irradiation, pulsed light, ultrasound). Thermal treatments are highly effective in achieving substantial log reductions (between 1 and 7 log_10_ reduction) of *L. monocytogenes* (see Table 5: Physical intervention strategies: Thermal); however, they may not be suitable for heat-sensitive products, such as cheese or vacuum-packed meats, due to potential changes in texture, color, flavor, or packaging integrity. For such products, non-thermal alternatives are increasingly preferred, as they offer gentler effects on food quality while still achieving notable 1–2 log_10_ reductions and sometimes complete elimination of *L. monocytogenes* (see Table 5; Physical intervention strategies: Non-thermal). Despite their benefits, some technologies remain costly, and certain methods, like irradiation, face barriers due to limited consumer acceptance.

Chemical interventions involve the use of approved additives, such as sodium lactate or sodium diacetate, and the use of organic acid. These substances help lower pH or create antimicrobial environments that inhibit *L. monocytogenes* proliferation. Under sufficient concentrations, this can also achieve non-thermal inactivation, but additives are not a substitute for a validated kill step [108,137]. Research has shown that while these interventions are effective in inhibiting growth, overuse may affect the sensory properties, such as increasing saltiness, or may lead to heat resistance in *L. monocytogenes* when used in combination with salt (see Table 5: Chemical intervention strategies). The use and approval of chemical preservatives also varies between countries. For example, sodium lactate (E325) is an approved food additive in many regions, but its use is not permitted in foods intended for infants and toddlers [102,103].

Biological interventions leverage natural antimicrobials, such as bacteriophages, bacteriocins, lactic acid bacteria, and essential oils, offering sustainable alternatives to physical or chemical interventions. These antimicrobials can be highly specific to combat *L. monocytogenes*, making them ideal for applications where clean-label or minimal processing is desired [138,139]. However, their effectiveness is often lower than that of physical or chemical methods, with reported reductions in *L. monocytogenes* ranging between 0.4 and 4.0 log_10_ or inhibition of growth for a short period of time (3–5 days) (see Table 5; Biological intervention strategies). Their efficacy can also depend on the food matrix. For example, bacteriophages are generally more effective in liquid products due to better distribution and contact with the bacterial cells [104,140]. Additionally, regulatory approval varies by country. For instance, nisin is a bacteriocin produced by certain strains of *Lactococcus lactis*. In Australia, the maximum limit of 12.5 mg/kg of nisin is permissible in processed meat products [105]. In the US, the limit is 5.5 mg/kg for RTE meats and poultry products [106]. However, in Europe and Canada, the nisin limit in RTE meat products is 25 mg/kg [100,107].

**Table 5:** Efficacy of the intervention strategies used to control *L. monocytogenes* in RTE foods.

Types of Intervention Strategies	Description	Product Type	Example of Efficacy	Reference	Limitation ^1^
Physical intervention strategies: Thermal					
Pasteurization using conventional heating	Use of thermal heat above 72 °C to destroy *L. monocytogenes*.	RTE deli turkey	1.95–3 log_10_ reduction at 93.3 °C for 2–5 min	[141]	High temperatures might affect the texture, flavor or nutritional quality. Possible issue with the packaging, as it will need to withstand high temperatures.
		RTE deli meats	3 log_10_ reduction within 266 min at 55 °C or 44 min at 60 °C or 23 min at 62.5 °C or 5 min at 65 °C	[142]	
		RTE chicken drumette	7 log_10_ reduction at 54 min and 28 min at 60 °C and 70 °C, respectively, or at 18 min and 19 min at 80 °C and 90 °C, respectively	[143]	
		Lean sausage	7 log_10_ reduction within 8.1 min at 68.9 °C	[130]	
		Fat sausage	7 log_10_ reduction within 8.4 min at 68.9 °C	[130]	
		Lean ham	7 log_10_ reduction within 6.3 min at 67.9 °C	[130]	
		Fat ham	7 log_10_ reduction within 5.8 min at 68.9 °C	[130]	
Pasteurization via infrared heating	Use of infrared radiation to kill *L. monocytogenes*.	Deli ham	0.75–1.85 log_10_ reductions when processed for 45–75 s	[144]	Limited penetration capability for complete destruction of the bacteria [145].
		Roast beef	3.8 log_10_ reductions when processed for 60–90 s	[144]	
		Turkey frankfurters	3.5, 4.3, and 4.5 log_10_ reductions at temperatures of 70 °C, 75 °C, and 80 °C for 82 s, 92 s, and 103 s, respectively	[146]	
		Sliced ham	4.16 log_10_ reduction after 50 s	[147]	
		Cooked chicken	3.7 log_10_ reduction 62 °C within 9.3 min; 4.9 log_10_ reduction at 68 °C within 5.2 min; 6.0 log_10_ reduction at 75 °C within 3.6 min	[145]	
Physical intervention strategies: Non-thermal					
High-pressure processing (HPP)	Use of high pressure (400–600 MPa) for microbial inactivation [148,149].	Fresh Hispánico-type cheese	3.8 log_10_ reduction at high pressure of 400 MPa for 3 min	[150]	High equipment cost and possible issue with the packaging, as it will need to withstand high pressure [151].
		Ripe Mahón cheese	1 log_10_ reduction at 400 MPa for 18 min	[150]	
		RTE cooked chicken	3.3 log_10_ reduction at pressure level of 600 MPa for 2 min	[152]	
		RTE sliced cooked ham	4 log_10_ reduction at 504 MPa for 3 min	[153]	
		Sliced mortadella	4 log_10_ reduction at 526 MPa for 3 min	[153]	
		Sliced dry cured ham (white pig)	3.9 log_10_ reduction at 600 MPa for 5 min	[154]	
		Sliced dry cured ham (Iberian pig)	1.9 log_10_ reduction at 600 MPa for 5 min	[154]	
		RTE cooked chicken	1.4 log_10_ reduction at 600 MPa for 2 min	[155]	
		Dry cured ham	0.92–3.92 log_10_ reduction at 450 MPa for 5 min	[156]	
		Dry cured ham	6.28–6.82 log_10_ reduction at 600 MPa for 5 min	[156]	
		Dry cured ham	5.26–7.96 log_10_ reduction at 750 MPa for 5 min	[156]	
		Mozzarella	3–4 log_10_ reduction at 400 MPa for 10 min	[157]	
		Mozzarella	Not detected at 500 MPa for 10 min	[157]	
		Smoked salmon	1.5 log_10_ reduction at 500 MPa for 10 min	[157]	
		Salchichon (dry cured sausage)	3.42 log_10_ reduction at 600 MPa for 8 min	[158]	
		Dry cured loin	3.08 log_10_ reduction at 600 MPa for 8 min	[158]	
		Bacon	1.68 log_10_ reduction at 593 MPa for 5 min at 4.0 °C	[159]	
		Smoked rainbow trout	1.0–1.82 log_10_ reduction at 500 MPa for 5 min at 4.4 °C	[160]	
		Smoked rainbow trout	1.2–2.73 log_10_ reduction at 500 MPa for 5 min at 4.4 °C	[160]	
Irradiation	Use of ionizing radiation to eliminate *L. monocytogenes* by destroying the DNA of *L. monocytogenes*, thereby leading to its inactivation.	Vacuum packed cooked ham	Achievement of FSC (10^2^ CFU) and zero tolerance at doses of 1 kGy and 2.5 kGy of e-beam radiation	[161]	Possible issue with consumer acceptance, and expensive [162].
		Chicken breast Adobo	Complete elimination at a minimum dose of 25 kGy of gamma irradiation with total irradiation time of 4.6 h	[163]	
Pulse light (PL)	Use of short flashes with wavelengths of 200–1100 nm, including UV light known as pulse light, which disrupts DNA transcription and replication in *L. monocytogenes* [164].	Cooked ham	2 log_10_ CFU/cm^2^ reduction at pulse light of 8.4 J/cm^2^	[101]	Limited penetration capability [101]. Higher fluences may lead to change in sensory quality [101].
		Cooked bologna	1 log_10_ CFU/cm^2^ reduction at pulse light of 8.4 J/cm^2^	[101]	
		RTE cured meat products	1.5–1.8 log_10_ CFU/cm^2^ reduction at pulse light of 11.9 J/cm^2^	[165]	
Ultrasound	Washing technique applied by using bath-type ultrasound between frequencies of 20–100 kHz to destroy and detach the microorganisms from the food surface [166,167,168,169].	Lettuce	0.42 log_10_ CFU/cm^2^ at 10 min treatment time	[170]	May increase lipid oxidation. Ultrasound parameters should be optimized for each product type [168].
		Lettuce	0.39 log_10_ CFU/cm^2^ at 30 min treatment time	[170]	
		Lettuce	0.40 log_10_ CFU/cm^2^ at 60 min treatment time	[170]	
Chemical intervention strategies					
Chemical additives	The application of chemical substances, referred to as preservatives (e.g., sodium diacetate, sodium lactate) to reduce pH, therefore establishing an environment unfavorable to pathogens.	Vacuum-packaged frankfurters	Inhibited the growth and increased the storage time to 120 days when sodium lactate and sodium acetate used together (all at 0.25%)	[109]	Affects the sensory properties of the product [171,172]. May lead to heat resistance of *L. monocytogenes* when used in combination with salt [173].
		Weiners	Prevented the growth during storage at 4.5 °C for 60 days when ≥3% sodium acetate and combination of ≥1% lactate plus ≥ 0.1% diacetate was used	[137].	
Use of organic acids	Use of GRAS (generally recognized as safe) organic acid, such as such as acetic acid, lactic acid, citric acid, malic acid, and peracetic acid, to disrupt cell function.	Lettuce	1.18 log_10_ CFU/cm^2^ when 2% acetic acid used for 10 min	[170]	May affect the organoleptic quality of the product due to residue organic acid left on the product and also due to organic acid-specific odor and taste
		Lettuce	1.87 log_10_ CFU/cm^2^ when 2% acetic acid used for 30 min	[170]	
		Lettuce	2.68 log_10_ CFU/cm^2^ when 2% acetic acid used for 60 min	[170]	
		Lettuce	1.21 log_10_ CFU/cm^2^ when 2% lactic acid used for 10 min	[170]	
		Lettuce	2.12 log_10_ CFU/cm^2^ when 2% lactic acid used for 10 min	[170]	
		Lettuce	2.99 log_10_ CFU/cm^2^ when 2% lactic acid used for 10 min	[170]	
		Lettuce	6.01 log_10_ CFU/g when 1% acetic used for 0.5 min	[174]	
		Lettuce	5.33 log_10_ CFU/g when 1% acetic used for 0.5 min	[174]	
		Lettuce	5.04 log_10_ CFU/g when 1% acetic used for 1 min	[174]	
		Lettuce	4.34 log_10_ CFU/g when 1% acetic used for 1 min	[174]	
Biological intervention strategies					
Use of lactic acid bacteria (LAB) starter cultures	LAB inhibit *L. monocytogenes* by (i) acidification (pH reduction), (ii) competitive exclusion for nutrients and niches, and (iii) producing antimicrobials (organic acids, hydrogen peroxide, bacteriocins, such as nisin) [175,176].	Cheese	1.48–4.16 log_10_ for lactococci 1.96–4.21 for lactobacilli	[177]	Should be used as a part of hurdle technology, as this singly will not ensure complete safety [178]. Strains used should be GRAS-approved or assigned Qualified Presumption of Safety (QPS) [178,179]. Food matrix might influence the survival or replication of *L. monocytogenes* and LAB [179].
		Gorgonzola cheese	Not detected in 25 g after addition of two strains of lactic acid bacteria, *Lactococcus lactis* subsp. *Cremoris* FT27.	[180]	
		Soft cheese	0.5–1 log_10_ CFU/g reduction when a mixture comprising three lactic acid bacteria was added	[181]	
		Dry-cured fermented sausage, “salchichon”	1.6–2.2 log_10_ CFU/g reduction after addition of *Lactobacillus sakei* 205	[182]	
Bacteriophages	Use of bacteriophages, which are viruses that infect and replicate within bacteria, thus killing the host [183]. E.g., phages such as A511 and P100 were effective in controlling *L. monocytogenes* in RTE foods [184].	Fresh-cut red delicious apples	0.4 log_10_ reduction after application of nisin and phage mixture	[185]	Efficiency is dependent on intrinsic and extrinsic parameters, such as phage concentration, food matrix, and storage conditions. More efficient in liquid products.
		Honeydew melons stored at 10 °C	2.0–4.6 log_10_ reduction after application of nisin and phage mixture	[185]	
		Cooked turkey	2.1 log_10_ CFU/cm^2^ reduction when PhageGuard Listex^TM^ P100 was used at 4 °C	[186]	
		Cooked turkey	1.5 log_10_ CFU/cm^2^ reduction when PhageGuard Listex^TM^ P100 was used at 10 °C	[186]	
		Roast beef	1.7 log_10_ CFU/cm^2^ reduction when PhageGuard Listex^TM^ P100 was used at 4 °C	[186]	
		Roast beef	1.7 log_10_ CFU/cm^2^ reduction when PhageGuard Listex^TM^ P100 was used at 10 °C	[186]	
		Cheese	0.7 log_10_ reduction by using the commercially available phage, ListShield	[187]	
		Smoked salmon	1 log_10_ reduction by using the commercially available phage, ListShield	[187]	
		Smoked salmon	0.85, 2.4, 2.75, 2.34, and 1.58 log_10_ reduction at 1, 5, 10, 15, and 30 days after application of bacteriophage P100	[188]	
		Hot dog	>2 log_10_ concentration reduction after addition of lactic acid bacteria	[189].	
Bacteriocins	Use of antimicrobial peptides (e.g., nisin) produced by certain bacteria, such as lactic acid bacteria, which attacks the cell membrane of the pathogen, resulting in damage to the membrane structure [190,191,192].	RTE sliced pork ham	2.83 log_10_ reduction by bacteriophage P100 (LISTEX^TM^ P100)	[193]	Higher concentrations of bacteriocin are required to inhibit growth [139]. Intrinsic properties of food will affect the stability of bacteriocin, which is unstable to pH changes [139]. Country-specific restriction in the use of nisin in food [100,104,105,106,107]. Should be used as a part of hurdle technology.
		Cooked ham	1.85 log_10_ reduction by 200 AU/cm^2^ nisin A (N200)	[194]	
		Cooked ham	1.80 log_10_ reduction by 200 AU/g nisin plus 1.8% potassium lactate	[194]	
		Cooked ham	4 log_10_ reduction by interleavers (containing mixture of enterocins, sakacin, nisin, potassium lactate)	[194]	
		Cooked ham	4 log_10_ reduction by interleavers (containing mixture of enterocins, sakacin, nisin, potassium lactate)	[194]	
		RTE Russian type salad	Not detected in 25 g until day 5 when 40 µg/g of enterocin AS-48 was used at 10 °C	[195]	
		RTE turkey ham	4 log_10_ CFU/g reduction at 0.4% and 0.5% nisin treatment after 7 and 14 days of storage, respectively.	[196]	
		Sliced dry cured ham (white pig)	0.80 log_10_ reduction when nisin was applied directly (200 AU/mL), and 3 log_10_ reduction after 60 days of storage	[154]	
		Sliced dry cured ham (Iberian pig)	1.24 log_10_ reduction when nisin was applied directly (200 AU/mL), and 3 log_10_ reduction after 60 days of storage	[154]	
		Smoked salmon	2, 3.4, 4.5, 4.25, and 4.25 log_10_ reductions at 1, 5, 10, 15, and 30 days after spraying with enterocin AS-48	[188]	
		Gorgonzola cheese	Not detected after addition of two strains of lactic acid bacteria, *Lactococcus lactis* subsp. *Cremoris* FT27	[180]	
		Soft cheese	0.5–1 log_10_ CFU/g reduction when a mixture comprising three lactic acid bacteria was added.	[181]	
		Dry-cured fermented sausage, “salchichon”	1.6–2.2 log_10_ CFU/g reductions after addition of *Lactobacillus sakei* 205	[182]	
		Sardinian dairy products	3–4 log_10_ CFU/g reductions when a mixture of lactic acid bacteria was added	[177]	
		Jben (a Moroccan fresh cheese)	1.46 log_10_ CFU/g reduction) in comparison to control after addition of enterocin OS1 (200 AU/g) after 15 days of storage at 8 °C	[197]	
Spices, herbs, and essential oils	Use of spices, herbs, and essential oil derived from plants (e.g., thyme, rosemary), to provide bioactive components, such as phenolic acids, terpenes, aldehydes, and flavonoids, which have antimicrobial properties that help damage the cellular structure of the pathogen [198].	Ham slices	2.5, 2.6, and 3.0 log_10_ reductions with addition of clove, rosemary, cassia bark, and licorice, and 2.9, 3.0, and 3.2 log_10_ reductions in MAP	[199]	May affect taste and aroma of products.
		Cheese (curd)	No growth observed at concentration of 2000 ppm (day 1–5)	[200]	
		Smoked Salmon	No growth observed at concentration of 4000 ppm (day 1–3)	[200]	
		Italian mortadella	Growth was 2.29–2.79 log_10_ CFU/g less in samples treated with thyme and rosemary compared to the control samples after 30 days of storage	[201]	

Note: ^1^ The limitations presented here are only overall limitations of the particular intervention strategies and are not specific to any of the studies mentioned.

#### 5.2.1. Challenges and Considerations in Implementing Intervention Strategies

Implementing effective safety interventions for *L. monocytogenes* in RTE foods present multiple challenges. The efficacy of these interventions depends largely on product specific factors, such as the type of RTE food, the product formulation, the properties of the food matrix, and the characteristics of the contaminating strains. By tailoring interventions to specific product types and processing environments, the food industry can enhance food safety while addressing the aforementioned challenges. This section focuses primarily on these factors influencing intervention effectiveness. Other considerations, such as consumer preference, clean label demand, and economic feasibility, are also important but are beyond the primary scope of this review.

(a) Influence of different types of RTE foods: Intervention strategies must be adapted depending on the characteristics of RTE foods, such as deli meats, cheeses, and salads. For example, thermal treatments above 72 °C are effective in destroying *L. monocytogenes* in deli meats without significantly affecting sensory quality [142]. However, for heat-sensitive products like RTE salads, such treatments can negatively impact texture and flavor. In these cases, non-thermal methods like high-pressure processing (HPP), which keeps the product temperatures below 45 °C, are more suitable [149,150]. Koutsoumanis et al. [127] reported that RTE salad can be processed at 400–600 MPa for 1.5–3.0 min at 4–8 °C to meet the microbiological criteria. Similarly, irradiation at 2.5 kGy may eliminate *L. monocytogenes* in RTE cooked meats but can cause undesirable odors, leading to consumer rejection [161]. This illustrates the importance of balancing microbial safety with sensory qualities, such as taste, texture, and appearance, which are critical for consumer acceptance [202].
(b) Influence of food matrix properties: The intrinsic properties of RTE food, such as pH, a_w_, fat content, and nutrient composition play a crucial role in determining the effectiveness of intervention strategies. For example, Verheyen et al. [203] showed that fat enhanced *L. monocytogenes* inactivation in emulsions but offered protection in gelled emulsions (mimicking processed fish products), likely due to differences in heat transfer and shielding effects. Similarly, studies using various meat products show that fat content and a_w_ can modulate microbial lethality, sometimes enhancing or limiting inactivation depending on the processing method [153,156,204]. In ground beef, fat reduced heat resistance at lower temperature (51.7 °C) but increased it when subjected at higher temperatures (57.2 °C and 62.8 °C) [204]. Furthermore, Bover-Cid et al. [156] compared two types of dry-cured ham during HPP treatment: one with a higher a_w_ (0.92) and lower fat content (14.25%), and the other one with a lower a_w_ (0.88) and higher fat content (33.26%). The former showed greater inactivation (5.26 log_10_ reduction at 750 MPa), while the latter was more resistant (0.92 log_10_ reduction at 450 MPa). Conversely, in cooked ham and mortadella, higher fat content was linked to lower HPP effectiveness [153]. Salt concentration, in combination with temperature, can also significantly influence *L. monocytogenes* survival and growth dynamics. In meat emulsions with 20% fat, different NaCl concentrations (2.5%, 5.0%, and 7.5%) produced markedly different growth outcomes depending on storage temperature (10, 20, and 30 °C) [205]. At higher temperatures (20–30 °C), lower salt levels (2.5% and 5.0%) allowed for faster growth, whereas 7.5% NaCl had an inhibitory effect [205]. However, at 10 °C, *L. monocytogenes* was able to grow even at 7.5% NaCl, reaching 6.8 log_10_ CFU/g after 12 days, indicating its ability to survive under cold, high-salt conditions [205]. Similarly, in a challenge test involving twelve RTE salad products, *L. monocytogenes* was able to grow in two types: tomato–cucumber without salt and lemon juice, and tahini salad at 4, 10, and 24 °C [206]. This was attributed to higher pH (>4.6) and release of nutrients from the tomato and cucumber [206]. In another study about Pinata (RTE Italian sausage), it has also been reported that the concentration of LAB and low a_w_ (0.91) and pH (5.8–5.9) acted as the key hurdles to the growth of *L. monocytogenes* [207]. These examples illustrate how fat content interacts with other matrix factors, such as a_w_, pH, and nutrient composition, to influence microbial survival, emphasizing the need for tailored interventions that consider the specific properties of each RTE product.
(c) Strain variability and stress resistance: Different *L. monocytogenes* strains exhibit varying degrees of tolerance to common stresses encountered in food processing and within the host, such as acid, osmotic, and thermal stress, as well as biofilm production [131,132,133,208,209]. The glutamate decarboxylase (GAD) system, a key acid-resistance mechanism, also varies between strains, with higher GAD activity correlating with improved survival in gastric juice [208,209]. Mechanistically, the *L. monocytogenes* GAD system comprises intracellular GAD (GAD_i_) and an antiport arm (GAD_e_), which are activated at different pH levels and differ by strain and medium, helping explain between-strain differences in acid tolerance. For example, when strains 10403S and LO28 were both exposed to pH 2.7, 10403S survived, whereas LO28 lost viability [210]. Notably, preliminary studies suggest that clinical *L. monocytogenes* isolates often possess significantly higher GAD activities compared to food isolates, indicating that strains more capable of causing human infection may be inherently more resistant to acidic conditions [208,211]. Additionally, clinical strains produced biofilm at higher incubation temperatures compared to strains isolated from food factories [209]. Consistent with this variability, Aryani et al. [212] reported D-values (time required at a specific temperature and condition to reduce the microbial population by one decimal) of twenty *L. monocytogenes* strains ranging from 9 to 30 min at 55 °C, 0.6 to 4.0 min at 60 °C, and 0.1 to 0.60 min at 65 °C, highlighting broad differences in thermal resistance. Similarly, Wang et al. [213] reported wide kinetic ranges across 33 *L. monocytogenes* strains, with maximum growth rates (µ_max_), lag times (λ), and (D_60_) time required for 1 log_10_ reduction at 60 °C ranging between 0.20 and 0.45 h^−1^, 0.24 and 3.36 h, and 0.52 and 3.93 min, respectively. They also showed that the mild acid adaptation (pH 5.5) increased heat resistance for most strains and produced survival curves with a shoulder during 60 °C inactivation, while leaving growth kinetics largely unchanged [213]. Heredia et al. [214] studied the inter-strain variability of twenty-six clinical and food *L. monocytogenes* isolates and concluded that strains from the meat category exhibited the lowest average pH_min_ (4.57), indicating potential acid adaptation. This inherent variability means that a universal approach to *L. monocytogenes* control may be inadequate, as highly resistant strains could persist despite interventions employed. Beyond strain-level variation, there are different serotypes of *L. monocytogenes*, out of which serotype 4b is associated with 50% of human outbreaks and serotypes 1/2a, 1/2b, and 1/2c are mostly isolated from food [68,71]. These differences in serotype distribution and virulence further underline that a universal approach to *L. monocytogenes* control may be inadequate, as certain serotypes and strains may persist or remain infectious despite interventions. A more nuanced understanding of both strain- and serotype-specific resistance is therefore critical to developing targeted and robust intervention strategies. A risk-based, virulence, and pathogenicity classification developed by the Joint FAO/WHO for *L. monocytogenes* should be considered similar to the approach used for Shiga toxin-producing *E. coli* (STEC) in food [215,216].
(d) Other considerations: Beyond technical efficacy, factors such as consumer perception and cost can significantly influence the adoption of intervention strategies. For example, consumer concerns about irradiation and chemical preservatives have driven demand for clean-label, minimally processed products [217,218,219,220]. This shift has encouraged the food industry to explore novel biocontrol approaches for managing *L. monocytogenes* in RTE foods, such as bacteriophages, bacteriocins, spices, herbs, and essential oils [177,181,186,200,202]. Likewise, advanced technologies like HPP and pulsed electric fields (PEFs), while effective, may be prohibitively expensive for small and medium-sized producers [221]. Although these factors are outside the main scope of this section, they remain important in determining the real-world feasibility of intervention implementation.

Overall, the successful implementation of *L. monocytogenes* intervention strategies in RTE foods requires a tailored, evidence-based approach. Factors such as product-specific characteristics, food matrix composition, and strain variability must be considered. The demand for minimally processed, clean-label products call for the application of biological interventions, where feasible. Balancing microbial safety with product quality remains a critical challenge for food producers and regulators, highlighting the importance of continued research and innovation in food safety management.

#### 5.2.2. Multi-Hurdle Approaches for Controlling *L. monocytogenes*

Controlling *L. monocytogenes* is particularly challenging due to its persistence in food processing environments and its ability to proliferate at refrigeration temperatures, even under modified atmospheric packaging. These challenges coupled with the limitations of single intervention have led to the development of multi-hurdle approaches that combine intervention strategies. Overall, studies consistently show that combining high-pressure processing (HPP) with natural antimicrobials, such as plant-derived compounds, bacteriocins, or bacteriophages, produces greater and more sustained *L. monocytogenes* reductions than when these treatments are applied individually [127,154,188,193,194,222,223]. While the magnitude of the benefit varies with product type, the general trend is that multi-hurdle treatments both accelerate pathogen inactivation and prolong the period before regrowth occurs. For instance, Bleoancă et al. [223] evaluated a combined treatment of thyme-derived natural antimicrobials with post-processing (HPP) in cheese. This combination resulted in a maximum reduction of 5.12 log_10_ CFU/g of *L. monocytogenes*, compared to only 1.68 log_10_ CFU/g when HPP was applied alone [223]. Similarly, Komora et al. [222] studied the impact of HPP (300 MPa) and bacteriophage P100 and pediocin PA-1 in fermented sausage, which resulted in 5 log_10_ reduction, completely eliminating *L. monocytogenes* immediately after processing. However, using these interventions individually did not eliminate the pathogen. Even the combination of HPP with P100 alone did not eliminate the pathogen even after 60 days of storage [222]. The combination of HPP and pediocin PA-1 did eliminate *L. monocytogenes*, but the effect was delayed by 72 h [222]. Figueiredo and Almeida [193] also found that combining nisin with bacteriophage P100 in RTE sliced pork ham resulted in a 3 log_10_ reduction of *L. monocytogenes* after 72 h of storage at 6–8 °C. These emphasize the synergistic effect between natural compounds and non-thermal inactivation methods.

Another study by Baños et al. [188] on smoked salmon fillets concluded that bacteriocin AS-48 alone reduced *L. monocytogenes* by 2-4.5 log_10_ CFU/cm^2^ during storage (below detection at day 10), while phage P100 alone achieved 0.85-2.75 log_10_ reductions. When combined, bacteriocin AS-48 and P100 maintained *L. monocytogenes* below detection (<10 CFU/cm^2^) from days 1-15, with only slight regrowth to 0.78 log_10_ CFU/cm^2^ by day 30 [188]. Similarly, Hereu et al. [154] concluded that the combined effect of HPP (600 MPa for 5 min) and nisin-activated polyvinyl film containing 200 AU/cm^2^ resulted in reductions of 5.9 log_10_ and 4.1 log_10_ in dry cured ham with a_w_ of 0.92 and 0.88, respectively. This is in contrast to the single treatment of HPP, which only resulted in 3.9 and 1.9 log_10_ reductions, respectively [154]. In another study by Jofre et al. [194], the combination of interventions (HPP and bacteriocins) not only resulted in the elimination of *L. monocytogenes* but also extended shelf life. When cooked ham was pressurized at 400 MPa for 10 min and then treated with interleavers (thin sheets placed between slices) containing bacteriocins, i.e., 200 and 200 AU/cm^2^ of enterocins (E200 and E2000), 200 and 200 AU/cm^2^ of sakacin (S200 and S2000), 200 AU/cm^2^ of nisin A (N), 1.8% potassium lactate (L), a combination of 200 AU/g nisin plus 1.8% of potassium lactate (NL), 4.2-4.5 log_10_ reductions were achieved, with S2000 yielding absence of *L. monocytogenes* in 25 g samples [194]. During storage, S200, N, and NL kept *L. monocytogenes* below 3 MPN/g for 45 days, and S2000, E200, and E2000 maintained this for an additional 15 days [194]. These findings demonstrate that while single interventions can be effective, combining strategies often provides greater and more consistent reductions in *L. monocytogenes*, providing hurdles that ensure food safety, extend shelf life, and maintain the sensory and nutritional quality of RTE foods.

Among the various multi-hurdle strategies, some approaches show greater practicality and promise for industry adoption based on effectiveness, cost, regulatory acceptance, and compatibility with existing processing lines. In current practice, processors commonly employ organic acid salts (e.g., potassium lactate, sodium diacetate) and nisin [224]. High-pressure processing (HPP) is widely adopted by many RTE meat producers due to its ability to inactivate *L. monocytogenes* without significant impact on product quality and its regulatory acceptance as a non-thermal listericidal treatment [225]. When combined with GRAS (Generally Recognized as Safe) antimicrobials, such as nisin or organic acids (e.g., potassium lactate), this strategy becomes even more robust. Bacteriophage P100, approved for use in several jurisdictions (e.g., US, EU, Australia), also presents a promising addition, particularly because it can be applied post-packaging via spraying or dipping without requiring major processing changes. However, standalone phage application often shows limited efficacy and is more effective when used in combination with HPP or bacteriocins [194]. In summary, HPP combined with bacteriocins, or organic acid-based preservatives is currently the most feasible multi-hurdle approach for broad industrial application in RTE foods due to its proven efficacy, regulatory clearance, and scalability. Other promising methods, like lactic acid bacteria biocontrol, natural preservatives, and active packaging systems, are emerging but may require further optimization, regulatory clarity, and cost–benefit evaluation before widespread implementation.

### 5.3. Future Studies

Despite significant advancements in understanding the risk of listeriosis and implementation of relevant control measures for RTE foods, further research is needed to enhance food safety outcomes. The effectiveness of these interventions can be strongly influenced by product-specific intrinsic factors, such as pH, a_w_, and fat content. These intrinsic properties can affect how *L. monocytogenes* grows, survives, or become inactivated under different processing conditions. Strain variability plays a critical role in determining the pathogen’s resistance to environmental and technological stresses [208,209,212]. However, existing studies have primarily focused on a limited range of RTE products and have used a narrow selection of *L. monocytogenes* strains [153,156,203,204,226], which can result in under- or overestimation of intervention efficacy.

To account for this variability, reduce uncertainty, and improve food safety decision making, researchers have increasingly turned to QMRA models. Several well-established models (e.g., [227,228,229] integrate predictive microbiology (Baranyi for growth, Bigelow/Weibull for inactivation, etc.) to simulate *L. monocytogenes* behavior and the impact of interventions, such as thermal processing and HPP. These models represent a significant advancement in QMRA by linking growth or inactivation kinetics of *L. monocytogenes,* but they are often based on data generated under controlled laboratory settings and may not reflect the full complexity of commercial food processing environments. In particular, accurate determination of growth and inactivation parameters under real-world conditions, including variability in temperature, pH, a_w_, and product composition, are critical to improving the reliability of predictive models. For instance, the predictive model for *L. monocytogenes* in seafood and meat products developed by Mejlholm and Dalgaard [230] includes the effects of 12 environmental parameters, including temperature, pH, a_w_, salt, CO_2_, smoke components, nitrite, acetic acid, benzoic acid, citric acid, diacetate, lactic acid, and sorbic acid [230,231,232]. It also accounts for microbial interaction with lactic acid bacteria via the Jameson effect [233]. Inclusion of such multi-factor growth models within the QMRA will not only strengthen risk assessments but will also support more targeted and effective *L. monocytogenes* control strategies in RTE foods. Importantly, the application of QMRA could extend beyond illness risk estimation to predicting recall risks and determining microbiologically safe shelf life. This is where the approach could differ from existing models. For example, Chen et al. [234] applied QMRA to estimate the likelihood of regulatory non-compliance and recall risk for smoked salmon, while Koutsoumanis et al. [30] incorporated spoilage modeling to guide shelf-life setting. Building on these concepts, future work should develop industry-relevant, QMRA based decision-support tools that (i) integrate predictive growth accounting for strain variability and key environmental factors (temperature, pH, a_w_, nitrite, organic acids, CO_2_), including microbial interaction via the Jameson effect; (ii) include inactivation models; and (iii) evaluate single and multi-hurdle interventions (e.g., HPP with bacteriocins/protective cultures) to simulate their combined impact on *L. monocytogenes* control, shelf life, and recall risk. Such tools would enable food businesses to proactively manage contamination, optimize product shelf life, ensure regulatory compliance, and minimize the likelihood of costly recalls, ultimately contributing to safer RTE food systems.

## 6. Conclusions

*L. monocytogenes* remains one of the most significant foodborne pathogens affecting RTE foods, posing serious risks to public health and food industry operations. Its ability to persist in food processing environments, form protective biofilms, and grow at refrigeration temperatures makes it particularly difficult to control. Despite strict regulatory requirements set for *L. monocytogenes* in RTE foods, it continues to be associated with food recalls, especially high-risk RTE products. Comparisons between Australian surveillance and recall data, and available data from the US, Europe, and Canada confirm that RTE foods are common vehicles for *L. monocytogenes* contamination. Although compliance rates in Australia remain high, occasional non-compliant samples (detection of *L. monocytogenes* in food) and ongoing listeriosis cases still occur, indicating ongoing risk. The increasing demand for minimally processed, clean label foods also present new challenges for managing the risk of *L. monocytogenes* in RTE foods, particularly where conventional interventions like pasteurization may compromise product quality.

This review highlights the need for intervention strategies to be tailored to the intrinsic properties of specific RTE foods and the strain variabilities of *L. monocytogenes* while also balancing safety and sensory quality. Although QMRA tools have been primarily used for prediction of probability of illness, there is a growing interest in their use for predicting shelf life and recall risk. Future research should prioritize the development of a robust, industry-relevant, QMRA-based decision support tool that integrates predictive growth and inactivation models capturing strain variability, key environmental factors (temperature, pH, a_w_, nitrite, organic acids, CO_2_), and microbial interactions (e.g., the Jameson effect). Unlike the existing tool, it will output the optimum shelf life and recall risk of RTE food based on the multi-factor growth and inactivation models. Such a tool would align with evolving regulatory standards and help businesses proactively manage *L. monocytogenes* risks across the RTE supply chain, thereby reducing recall associated losses. By advancing these models and strengthening food safety practices across the RTE food supply chain, it will be possible to reduce public health risks, minimize product recalls and support safer food systems globally. Overall, controlling *L. monocytogenes* in RTE foods requires a comprehensive and adaptive approach that integrates scientific innovation, industry collaboration, and predictive modeling to achieve safer RTE food systems and protect public health.

## Figures and Tables

**Table 1:** Regulatory classification of RTE foods by *L. monocytogenes* growth potential and risk level across regions.

Category of RTE Foods	Regulatory Thresholds ^(1)^				Examples	Rationale
	Region	pH	a_w_	Reference		
High risk/Category 1	Australia	≥4.4	NA ^(2)^	[99]	Soft cheeses (Brie, Feta, Ricotta), deli meats, smoked seafood, cooked prawns, pre-packaged salads, fresh-cut fruit	High moisture and near-neutral pH allow *L. monocytogenes* to grow during storage
		-	≥0.92			
		≥5.0	≥0.94			
	Codex	≥4.4	-	[27]		
		-	≥0.92			
		≥5.0	≥0.94			
	Canada	≥4.4	-	[97]		
		-	≥0.92			
		≥5.0	≥0.94			
	Europe	>4.4	-	[96]		
		-	>0.92			
		>5.0	>0.94			
	US	>4.4	-	[16]		
		-	>0.92			
Low risk/Category 2	Australia	<4.4	-	[99]	Pickled vegetables, dry crackers, hard cheeses, jam, canned soups, fermented meats (salami, pepperoni), chocolate, vinegar foods	Low pH and/or water activity inhibit *L. monocytogenes* growth
		-	<0.92			
		<5.0	<0.94			
	Codex	<4.4	<0.92	[27]		
		<5.0	<0.94			
	Canada	<4.4	-	[97]		
		-	<0.92			
		<5.0	<0.94			
	Europe	≤4.4	-	[96]		
		-	≤0.92			
		≤5.0	<0.94			
	US	≤4.4	-	[16]		
		-	≤0.92			

^(1)^ Thresholds indicate the conditions under which *L. monocytogenes* is not expected to grow. For instance, Codex Alimentarius Commission guidelines CAC/GL 61-2007 states that a food is considered low-risk if it satisfies any of the following criteria: pH ≤ 4.4, regardless of a_w_; a_w_ ≤ 0.92, regardless of pH; pH ≤ 5.0 and a_w_ ≤ 0.94 (combined). Foods exceeding one or both of these limits are considered high-risk. The application of these thresholds may vary slightly by region, but they follow similar risk-based frameworks; ^(2)^ NA means not applicable.

**Table 2:** Summary of food recall incidents due to *L. monocytogenes* and other microbial contaminants.

Country	Year of Recall	Total Number of Recall Events	Recalls Incidents Due to Microbiological Contamination	Recalls Due to *L. monocytogenes*	Reference
Australia	2020–2024	446	90 (20.2%)	28 (31.1%)	[19]
Canada	2018–2023	821	316 (38.5%)	NA *	[21]
EU ^+^	2013–2022	11,684	6510 (55.7%)	654 (10.0%)	[20,121]
US	2012–2021	1093	213 (19.4%)	92 (43.2%)	[18]

NA * = not available. The Government of Canada recall portals (Canada Food Inspection Agency/Health Canada) list events by hazard category but did not publish an official, consolidated count of recalls by specific pathogen (e.g., *Listeria monocytogenes*) for 2018–2023. EU ^+^: Values are iRASFF notification counts (not consolidated recall counts).

**Table 3:** Prevalence data for *L. monocytogenes* in RTE foods in Australia.

Agency	Products	Year	Total no. of Samples	No. of Positive for *L. monocytogenes*	Positive Samples (%)	Reference
FSANZ	Cooked prawns	2003	230	4	2.0	[23]
NSW Food Authority	Packaged sliced RTE meats	2008	154	6	3.9	[24]
NSW Food Authority	RTE meat products (including poultry)	2011–2012	NA *	2	2.0	[26]
ACT Health Service	RTE sandwiches, rolls, and baked goods	2014–2016	NA	1	0.4	[25]

* NA means not available.

**Table 4:** Microbiological criteria for *L. monocytogenes* in RTE foods.

Region	Regulation/Document Name	Microbiological Criteria *		Reference
		High Risk/Category 1	Low Risk/Category 2	
Australia	Schedule 27 of the Australia New Zealand Food Standards Code	Absence in 25 g	≤100 CFU/g	[99]
Canada	Policy on *L. monocytogenes* in RTE foods, 2023	Absence in 25 g	≤100 CFU/g	[97]
Codex	CXC/GL 61-2207	Absence in 25 g	≤100 CFU/g	[27]
European Union	Regulation (EC) No. 2073/2005	Absence in 25 g	≤100 CFU/g **	[96]
US	US FDA Compliance policy guide (CPG), Article 1, Sec 555.320	Absence in 25 g	Absence in 25 g	[129]

* Growth parameters are defined in Table 1; ** absence in 25 g for RTE foods intended for infant and medical foods.

## Data Availability

The original contributions presented in the study are included in the article. Further inquiries can be directed to the corresponding author.
